# Regulation of connexin 43 by interleukin 1β in adult rat cardiac fibroblasts and effects in an adult rat cardiac myocyte: fibroblast co-culture model

**DOI:** 10.1016/j.heliyon.2019.e03031

**Published:** 2019-12-30

**Authors:** Lisa McArthur, Alexandra Riddell, Lisa Chilton, Godfrey L. Smith, Stuart A. Nicklin

**Affiliations:** a Institute of Cardiovascular and Medical Sciences, College of Medical, Veterinary and Life Sciences, University of Glasgow, UK; b College of Public Health, Medical and Veterinary Sciences, James Cook University, Townsville, Queensland, Australia

**Keywords:** Cell biology, Molecular biology, Cell culture, Membrane, Gene expression, Cardiovascular system, Myofibroblast, Inflammation, Heterocellular coupling, Cardiac myocyte contraction, Action potential

## Abstract

Connexin 43 expression (Cx43) is increased in cardiac fibroblasts (CFs) following myocardial infarction. Here, potential mediators responsible for increasing Cx43 expression and effects of differential CF phenotype on cardiac myocyte (CM) function were investigated. Stimulating adult rat CFs with proinflammatory mediators revealed that interleukin 1β (IL-1β) significantly enhanced Cx43 levels through the IL-1β pathway. Additionally, IL-1β reduced mRNA levels of the myofibroblast (MF) markers: (i) connective tissue growth factor (CTGF) and (ii) α smooth muscle actin (αSMA), compared to control CFs. A co-culture adult rat CM:CF model was utilised to examine cell-to-cell interactions. Transfer of calcein from CMs to underlying CFs suggested functional gap junction formation. Functional analysis revealed contraction duration (CD) of CMs was shortened in co-culture with CFs, while treatment of CFs with IL-1β reduced this mechanical effect of co-culture. No effect on action potential rise time or duration of CMs cultured with control or IL-1β-treated CFs was observed. These data demonstrate that stimulating CFs with IL-1β increases Cx43 and reduces MF marker expression, suggesting altered cell phenotype. These changes may underlie the reduced mechanical effects of IL-1β treated CFs on CD of co-cultured CMs and therefore have an implication for our understanding of heterocellular interactions in cardiac disease.

## Teaser

Cell biology; Molecular biology; Cell culture; Membrane; Inflammation; Gene expression; Cardiovascular system; Myofibroblast; Inflammation; Heterocellular coupling; Cardiac myocyte contraction; Action potential

## 1 Introduction

Cardiac myocytes (CMs) are responsible for cardiac contraction and therefore are undoubtedly the most important cell type in the heart. However, cardiac fibroblasts (CFs) contribute to a large proportion of the cellular composition and have a major role in the heart by facilitating the turnover of the extracellular matrix (ECM). Following cardiac insult, CFs proliferate in mass and undergo a phenotypic switch to become myofibroblasts (MFs) [1]. These cells have a contractile phenotype, developing the expression of α-smooth muscle actin (αSMA), and secrete excessive volumes of ECM components, resulting in typical fibrosis seen after injury [2]. This contributes to cardiac dysfunction and dramatically increases the proarrhythmic potential of the heart, by creating structures that disrupt the conduction through the heart and predispose the conduction pathway to re-entry [3].

In the healthy heart, gap junctions (GJs), composed of connexin (Cx) proteins, are mainly present at the intercalated discs between neighbouring CMs and assist in rapid electrical conduction through the myocardium. Reduced and/or disorganised Cx43 (the main cardiac connexin) expression in CMs has been demonstrated in many cardiomyopathies [4, 5]. In contrast, CFs isolated from experimental models of myocardial infarction (MI) hearts, have been demonstrated to have augmented Cx43 levels in comparison to CFs from healthy hearts [6]. Interestingly this enhanced expression was also apparent in CFs from the area of infarct versus remote, noninfarcted regions [7]. The transfer of calcein dye between CMs and CFs *in vitro* [8, 9, 10] and *in situ* [11] suggests the presence of GJs between cell types. Additionally, electrical signals have been shown to propagate from one band of CMs to another when they were separated by CFs over distances of up to 300 μm in a culture model [12], demonstrating the ability of CFs to passively conduct electrical impulses.

Co-culture studies, mainly using cells from a neonatal origin, have suggested CFs can enhance the risk for arrhythmias involving both paracrine mediators and direct cell-to-cell interactions [13, 14, 15]. For instance, studies have reported a rise in the maximal diastolic potential (MDP; the most negative point of the action potential (AP))/resting membrane potential (RMP) of CMs [15, 16] as well as a reduction in conduction velocity (CV) [13, 15, 16] and enhanced spontaneous depolarisations [17] in co-cultures in comparison to CMs alone. Greater expression of Cx43 in CFs, seen in CFs from MI hearts, could result in enhanced coupling between CFs and CMs consequently enhancing the effects of CFs on CMs. Indeed, lentiviral (LV) vector-mediated silencing of Cx43 in CFs, diminished the rise in CM MDP and slowing of CV caused by CFs [13].

Interestingly, conditioned media from CFs from MI hearts induced a significant reduction in the CV and AP duration (APD) of rat neonatal cultures, whereas conditioned media from CFs isolated from healthy hearts had no effect [6]. Additionally, transforming growth factor (TGF) β1, a key driver of CF to MF transition [18], was shown to enhance the proarrhythmic nature of CFs on CMs in a neonatal cell culture model, by causing depolarisation of the RMP of CMs and promoting increased ectopic activity [19]. These effects in neonatal preparations were attributed to direct heterocellular coupling [19]. Furthermore, the ability of CFs to trigger spontaneous AP generation in CMs was shown to be dependent on αSMA expression in CFs [20]. Together these studies suggest the MF phenotype is able to cause detrimental effects on CMs in comparison to normal CFs.

Here, potential factors which may be responsible for the enhanced Cx43 expression in CFs found following MI were investigated. Interleukin (IL)-1β was found to enhance Cx43 levels, therefore the IL-1β pathway was dissected to establish the mechanism for this upregulation. Furthermore, untreated and IL-1β treated CFs were investigated for their effects on CM contraction and electrical activity in an adult CM: CF co-culture model.

## 2 Materials and methods

Expanded methods are available online, including full immunofluorescence and immunoblotting protocols.

### 2.1 Isolation and culture of rat adult ventricular cardiac fibroblasts and myocytes

Adult male Wistar rats (200–300g, Envigo, UK) were housed under 12 h light/dark cycles at ambient temperature with *ad lib* provision of standard chow and drinking water. Animals were killed by Schedule 1 according to the UK Animals (Scientific Procedures) Act 1986 and experiments approved by the University of Glasgow's Animal Welfare and Ethnical Review Board (AWERB). Hearts were removed from the thorax and lungs and placed in ice-cold solution. ADS buffer (composed of 116.4 mM NaCl, 20.0 mM HEPES, 1.0 mM NaH_2_PO_4_, 5.6 mM Glucose, 5.4 mM KCl, 0.8 mM MgSO_4_: pH 7.4) was used for CF isolation and heparinised Krebs-Henseleit (KH) solution (composed of 120.0 mM NaCl, 20.0 mM HEPES, 5.4 mM KCl, 0.5 mM NaH_2_PO_4_, 3.5 mM MgCl_2_.6H_2_0, 20.0 mM Taurine, 10.0 mM Creatine and 11.0 mM Glucose (Anhydrous): pH 7.4) used for CM isolation.

CF cultures were obtained by an enzymatic digestion method of the ventricular muscle, modified from previous published protocols [21, 22]. At the end of the digestion, cells were then pelleted and cultured in Dulbecco's Modified Eagle Medium: Nutrient Mixture F-12 (DMEM/F-12) supplemented with 10 % (v/v) foetal bovine serum (FBS), 100 I.U/mL penicillin and 100 μg/mL streptomycin. Culture media was replaced after 2–3 h. Adult rat CMs were isolated using a Langendorff perfusion set up similar to methods described elsewhere [23]. Expanded CF and CM isolation procedures are described in the supplementary material.

Where reduced serum media was required, DMEM/F-12 containing 0.5 % FBS, 100 I.U/mL penicillin and 100 μg/mL streptomycin was used. All plastics for CF culture were coated with poly-L-lysine.

### 2.2 Cardiac fibroblast stimulation

CFs were quiesced in reduced serum DMEM/F-12 (0.5 % FBS) for 24 h prior to stimulation and subsequent stimulations performed in this media. Cells were then reintroduced into culture DMEM/F-12 (10% FBS) or treated with 500 nM angiotensin (Ang) II (Phoenix Pharmaceuticals, Germany), 10 ng/mL recombinant human TGFβ1 (R & D systems, USA), 10 ng/mL recombinant human tumor necrosis factor (TNF)α (Peprotech, UK), 10 ng/mL recombinant human IL-1β (R & D systems), 10 ng/mL recombinant rat IL-1α (Peprotech) and 50 ng/mL recombinant human IL-6 (with or without 50 ng/mL recombinant human soluble IL-6 receptor α [sIL-6Rα]; both purchased from Peprotech). Cx43 mRNA (ThermoFisher Scientific, assay ID: Rn01433957_m1) was then measured 24 h following stimulation.

A range of IL-1β pathway inhibitors were utilised to dissect the IL-1β pathway. Firstly, 10 ng/mL IL-1β and recombinant human IL-1 receptor antagonist (IL-1Ra [Peprotech]) at 0.25, 0.5 or 1 μg/mL were added simultaneously to the cells. In a subset of experiments, a NF-κB inhibitor, SC-514 (100 μM [Cayman Chemical, USA]) was added 30 min prior to the addition of 10 ng/mL IL-1β. Furthermore, cells were incubated with inhibitors of p38 MAPK (SB203580 [InvivoGen, UK]; 10 μM), JNK (SP600125 [InvivoGen]; 20 μM) and MEK1 (AZD6244 [Selleckchem, USA]; 10 μM) for 1 h before IL-1β stimulation. As a vehicle control, 0.1% DMSO was used and all stimulations were performed for 24 h. Cx43 mRNA and protein expression were examined by qRT-PCR and western blotting respectively.

TGFβ1 is a well-established mediator of fibroblast to MF transdifferentiation [18, 24, 25]. Conversely, IL-1β has been reported to inhibit TGFβ1-mediated MF transdifferentiation in cardiac, dermal and lung fibroblasts [18, 26, 27]. Therefore, to identify the phenotype of IL-1β treated CFs, cells were stimulated with 10 ng/mL IL-1β, 10 ng/mL TGFβ1 or with a combination of both for 24 h and the mRNA expression of the MF markers, CTGF (assay ID: Rn01537279_g1) and αSMA (assay ID: Rn01759928_g1), examined.

### 2.3 Co-culture model

CFs were seeded at 5 × 10^4^ cells/35 mm glass bottomed petri dish and cultured for 48 h to allow the cells to reach a confluent monolayer. Cells were then serum starved for 24 h prior to the addition of 1 × 10^4^ CM rods. In experiments that involved the stimulation of CFs with 10 ng/mL IL-1β, CFs were serum starved for 24 h before stimulation. Cells were then washed once with sterile PBS prior to the addition of 1 × 10^4^ CM rods. CMs were seeded at the same density on laminin coated plates. CMs for calcein dye transfer and CM contraction experiments were added to CF monolayers in phenol red, reduced serum DMEM/F-12 whereas cells for CM AP measurements were maintained in phenol red-free, reduced serum DMEM/F-12. Co-cultures were fixed on completion of experiments for vimentin immunofluorescence.

### 2.4 Calcein dye transfer studies

The presence of functional GJs between CFs and CMs was assessed by imaging the transfer of calcein from preloaded CMs to an underlying monolayer of CFs. Freshly isolated CMs were loaded with 5 μM calcein acetoxymethyl ester (AM) made up in reduced serum DMEM/F-12 and incubated for 30 min at 37 °C. CMs were then washed three times in PBS before the addition of 1 × 10^4^ CM rods to each dish. Images were taken on the on the LSM 510 Meta laser scanning confocal microscope using the Argon/2 (488) laser, immediately and 20 h following CM addition, both focusing on the CM and CF layer to detect the calcein location. The laser intensity was increased at the 20 h time point due to the calcein fluorescence fading over time and to detect the transfer of calcein to the underlying CF layer.

### 2.5 Contraction measurement

CM contraction measurements were recorded following 24 h of culture while cells were maintained at 5 % CO_2_, 37 °C and in reduced serum DMEM/F-12 for the duration of the recordings. Cells were field stimulated at 1 Hz and the threshold voltage of each cell determined by increasing the voltage by 1 V increments until the cell started to respond. CMs were paced at 30 % above their threshold for 1 min before images were taken at 100 frames per second (fps) for 10 s using HCImage Live software. Typically, the contraction of 13 cells per condition in one experiment was recorded. The individual images were then stacked to construct a video of CM contraction and analysed on Image J using a macro (kind gift from Dr. Francis Burton [University of Glasgow]) to determine contraction duration (CD). The duration from the point of 50% contraction to 50% of the relaxation (CD50) was used as an indication of CD.

### 2.6 Action potential measurements

AP recordings were taken from CMs that had been freshly isolated and from CMs that had been cultured for 24 h. Phenol red can produce fluorescence [28] potentially generating artefacts in the AP traces so all recordings were performed in reduced serum DMEM/F-12 absent of phenol red. FluoVolt™ membrane potential kit (ThermoFisher Scientific) was used to detect changes in the CM membrane potential. AP measurements were performed at 37 °C within a darkened room. Cells were field stimulated at 1 Hz and using 40 V pulses and allowed to acclimatise for 5 min prior to the first recording. Next, 470 nm light used to excite the FluoVolt™ within the CMs and changes in membrane potential detected by CellOPTIQ software (Clyde Biosciences, UK) over a 10 s period. APs were analysed using the CellOPTIQ software, where APs were averaged and the average TRise (time taken from 10-90 % depolarisation) and APD_80_ values recorded for each cell.

### 2.7 Statistical analysis

All experiments were performed at least three times. Data from qRT-PCR experiments is represented as RQ ± rq_max_, where rq_max_ is indicative of the standard error of the mean (SEM) and is calculated from the ΔC_T_ value from each experiment. All other data is represented as mean ± SEM. For single CM data, statistical analysis was performed on the mean values for each heart. A Student's unpaired, two-tailed t-test was performed where there were two conditions. A repeated measures one-way analysis of variance (ANOVA) with a Dunnett's or Tukey's multiple comparisons test was performed where there were three or more conditions. All statistical analysis was performed using Graphpad Prism 4 software (GraphPad Software Inc., California, USA) and a *P* value < 0.05 was considered statistically significant.

### 2.8 Data availability

The datasets generated during and/or analysed during the current study are available from the corresponding author on reasonable request.

## 3 Results

**Figure 1:**
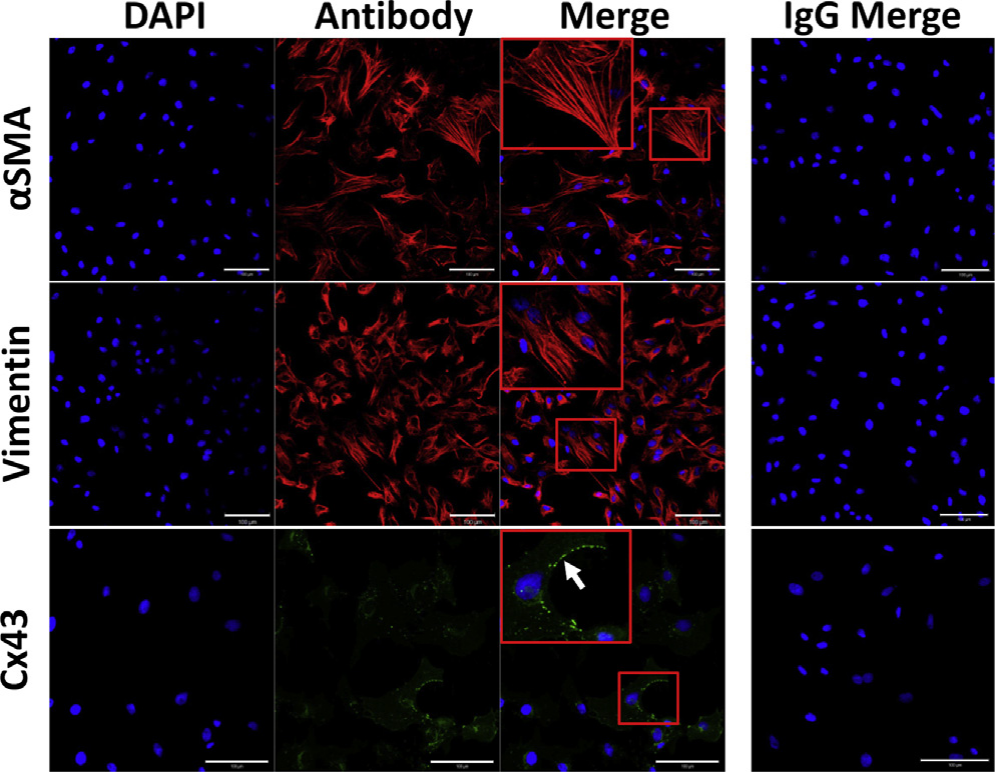
Immunolocalisation of αSMA, vimentin and Cx43 in passage two CFs. Immunostaining of αSMA (red: mouse monoclonal anti-αSMA [clone IA4]), vimentin (red: mouse monoclonal anti-vimentin [clone LN-6]) and Cx43 (green: rabbit polyclonal anti-Cx43) was performed on cells grown on glass for 48 h (*n=* 3). All images were normalised to the individual IgG shown down the right hand side. Nuclei were counterstained with DAPI (blue). Areas surrounded by a red box are magnified within the image and the white arrow denotes punctate expression of Cx43 at the cell membrane. Images were taken at x25 magnification. Scale bar represents 100 μm.

Passage 2–4 CFs were utilised throughout this study. By passage 2, CFs had a high expression of αSMA, suggesting differentiation of CFs to MFs in culture (Figure 1). CFs also expressed the fibroblast marker, vimentin, and Cx43 at the cell membrane (Figure 1).

### 3.1 IL-1β stimulation of CFs promotes enhanced Cx43 mRNA and protein levels

**Figure 2:**
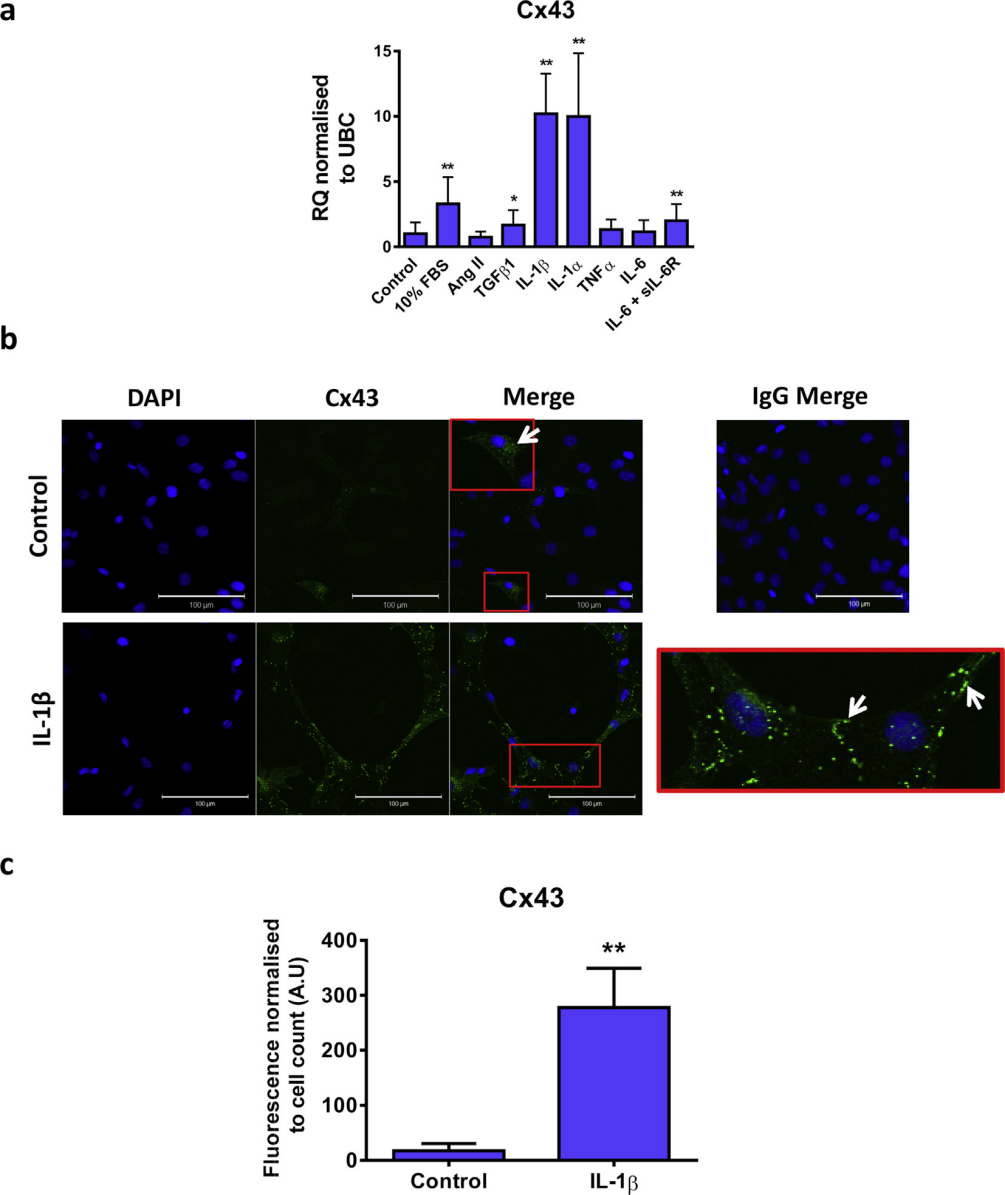
Effect of proinflammatory mediators on Cx43 expression in CFs. (A) Quiesced cells were stimulated with 10% FBS, Ang II (500nM), TGFβ1, IL-1β, IL-1α, TNFα (all 10ng/mL) or IL-6 in the presence or absence of the sIL-6R (both 50 ng/mL) for 24 h. Cells maintained in 0.5% FBS media were used as a control. Data represented as relative quantification (RQ) ± rq_max_ normalised to UBC (n = 3). *P < 0.05, **P < 0.01, ***P < 0.001 vs. control (one-way repeated measures ANOVA with Dunnett's multiple comparison test). (B) Immunostaining of Cx43 (green) in CFs maintained in 0.5% FBS media (control) or treated with 10ng/mL IL-1β (*n=*3). Nuclei were counterstained with DAPI (blue). Images were normalised to an IgG control shown in the upper right panel. The areas surrounded by a red box are expanded within the merged image or in the lower right panel for control and IL-1β stimulated cells respectively. White arrows indicate punctate Cx43 expression (green: rabbit polyclonal anti-Cx43). Images were taken at x25 magnification. Scale bar represents 100 μm. (C) Quantification of immunofluorescence normalised to the total cell number present in the images. At least 2 images per condition were analysed in each of the 3 independent experiments. **P < 0.01 vs. control (unpaired, two tailed t-test).

Previous studies have shown that CFs from infarcted hearts have enhanced Cx43 expression [6, 7]. As proinflammatory/profibrotic mediators are also augmented in cardiac disease [29, 30, 31], the effect of a range of proteins, including Ang II, TGFβ1, IL-1β, IL-1α, TNFα, IL-6 and IL-6 in combination with the sIL-6R on Cx43 mRNA expression was examined in CFs (Figure 2). Cx43 mRNA expression in CFs reintroduced into culture media (10 % FBS) following serum starvation was shown to be significantly greater than in control cells (relative quantification (RQ): 3.3 ± 2.1; *P <* 0.001; Figure 2A). Stimulation of CFs cultured in the absence of FBS with TGFβ1, IL-1β, IL-1α or IL-6 in combination with the sIL-6R induced a significant upregulation of Cx43 mRNA expression (RQ: 1.7 ± 1.1 [*P <* 0.05], 10.2 ± 3.1 [*P <* 0.001], 10.0 ± 4.9 [*P <* 0.001] and 2.0 ± 1.3 [*P <* 0.01] respectively; Figure 2A). Conversely, there were no significant changes in Cx43 mRNA following stimulation with Ang II, TNFα or IL-6 alone (RQ: 0.7 ± 0.4, 1.3 ± 0.8, 1.2 ± 0.8 respectively; Figure 2A).

IL-1β is one of the earliest cytokines to be detected in the plasma of MI patients [32], therefore its effects on CF function are particularly interesting. As both isoforms of IL-1 induced a similar increase in Cx43 mRNA, and are known to have indistinguishable actions [33], only IL-1β was investigated further. Cx43 protein expression was found to be significantly enhanced, particularly at the cell membrane (Figure 2B), by IL-1β treatment as detected by immunofluorescence (P < 0.01; Figures 2B and 2C).

**Figure 3:**
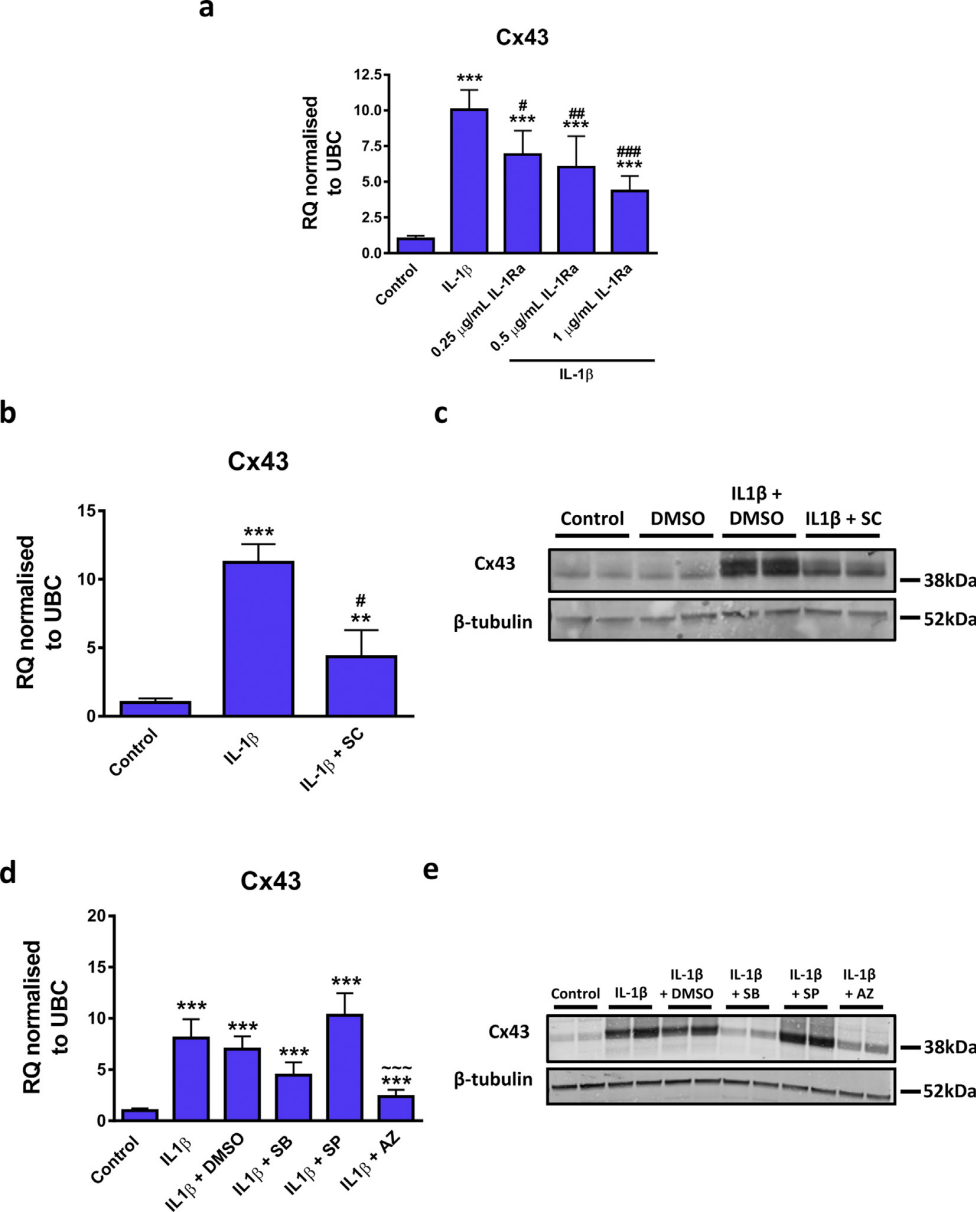
Effect of IL-1β pathway inhibitors on IL-1β mediated up-regulation of Cx43 mRNA expression in CFs. (A–E) Quiesced cells were stimulated with 10 ng/mL IL-1β for 24 h and inhibitors added at (A) the same time or (B and C) 30 min (D and E) to 1 h prior to stimulation. Cells maintained in 0.5% FBS were used as a control and 0.1% DMSO used as a vehicle control where required. (A, B and D) Cx43 mRNA data is represented as relative quantification (RQ) ±rq_max_ normalised to UBC. (C and E) Representative western blot showing Cx43 and β-tubulin control expression in replicate wells. The concentration of inhibitors used were (A) 0.25, 0.5 or 1 μg/mL human recombinant IL-1β receptor antagonist (IL-1Ra) (*n=* 3), (B and C) 100 μM SC-514 (SC) (*n=* 3), (D and E) 10 μM SB203580 (SB), 20 μM SP600125 (SP) or 10 μM AZD6244 (AZ) for inhibition of p38 MAPK, JNK or MEK1/2 respectively (*n=* 5 for mRNA and *n=* 3 for protein). **P < 0.01, ***P < 0.001 *vs.* control; ^#^*P <* 0.05, ^##^*P <* 0.01, ^###^*P <* 0.001 *vs.* IL-1β, ~~~ *P <* 0.001 *vs.* IL-1β + DMSO (one-way repeated measures ANOVA with Tukey's multiple comparison test). Full-length blots of panels C and E are presented in Supplementary Fig. S1 and S2, respectively.

Next, the mechanism by which IL-1β upregulated Cx43 expression was investigated. IL-1β-mediated upregulation of Cx43 mRNA was dose-dependently inhibited by the IL-1Ra (Figure 3A), indicating signalling through the IL-1R. Furthermore, as NF-κB is known to be an important regulator of IL-1β actions on CFs [34], the specific NF-κB inhibitor, SC-514 [35,36], was utilised. SC-514 significantly inhibited the IL-1β-mediated increase in Cx43 mRNA levels (Figure 3B). Similar to immunofluorescence data (Figures 2B and 2C), IL-1β appeared to increase Cx43 protein when detected through western blot (Figure 3C), an effect blunted in the presence of SC-514 (Figure 3C). Additionally, inhibition of MEK1 via the inhibitor AZD6244 was demonstrated to significantly inhibit Cx43 mRNA upregulation by IL-1β at the mRNA level, whereas there was no effect following inhibition of p38 MAPK with SB203580 or JNK with SP600125 (Figure 3D). Furthermore, the increase in Cx43 protein (Figure 3E) was blunted in the presence of SB203580 (Figure 3E) and AZD6244 (Figure 3E), whereas there was no inhibitory effect of the JNK inhibitor SP600125 (Figure 3E).

### 3.2 IL-1β and TGFβ1 have differential effects on the myofibroblast markers, αSMA and connective tissue growth factor (CTGF)

**Figure 4:**
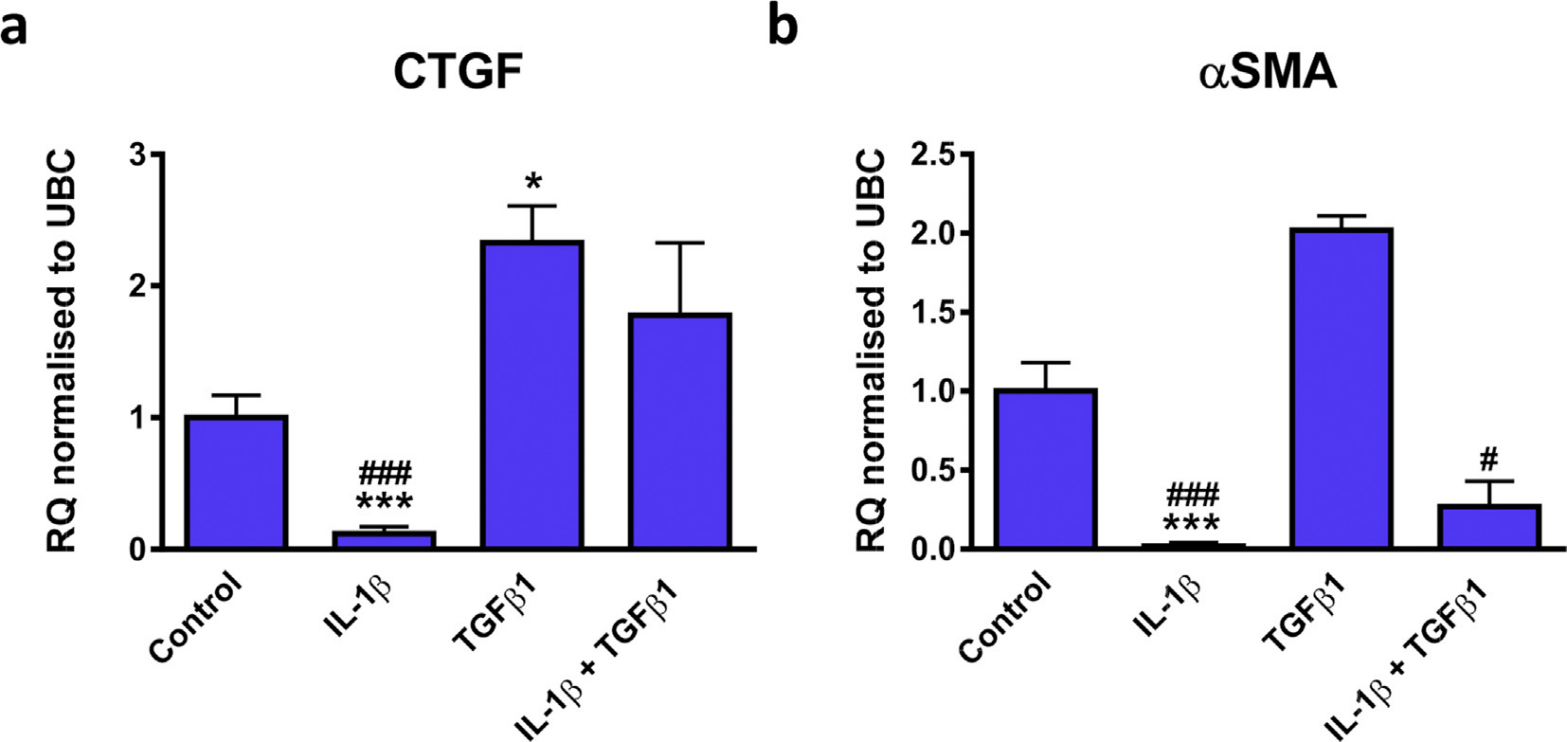
Effect of IL-1β or TGFβ1 alone or in combination on the mRNA expression of CTGF and αSMA in CFs. Quiesced cells were stimulated with IL-1β, TGFβ1 or IL-1β plus TGFβ1 (all at 10 ng/mL) for 24 h. Cells maintained in 0.5% FBS media were used as a control. mRNA levels of (A) CTGF or (B) αSMA was examined. Data represented as relative quantification (RQ) ± rq_max_ normalised to UBC (*n=*3). *P < 0.05, ***P < 0.001 *vs.* control; ^#^*P <* 0.05, ^###^*P <* 0.001 *vs.* TGFβ1 (one-way repeated measures ANOVA with Tukey's multiple comparison test).

Many mediators have been reported to induce fibroblast to MF transition, one of which is TGFβ1 [18,37]. Interestingly, IL-1β has been shown to inhibit TGFβ1-mediated MF differentiation of cardiac, dermal and lung fibroblasts [18, 26, 27]. Here we examined the effects of treatment of CFs with IL-1β and TGFβ1 alone or in combination on the expression of MF markers. As expected, IL-1β stimulation was found to have predominantly opposing actions on CTGF and αSMA gene expression to TGFβ1 (Figure 4). For instance, IL-1β promoted an 8 and 59 fold reduction in CTGF (RQ: 0.12 ± 0.05, *P <* 0.001; Figure 4A) and αSMA (RQ: 0.02 ± 0.02, *P <* 0.001; Figure 4B) levels, respectively. Conversely, TGFβ1 induced a significant up-regulation of CTGF (RQ: 2.3 ± 0.3, *P <* 0.05) but no significant change in αSMA expression (RQ: 2.0 ± 0.1). αSMA expression in IL-1β and TGFβ1 co-stimulated cells was significantly reduced in comparison to cells treated with TGFβ1 alone (RQ: 0.3 ± 0.16 *vs.* 2.0 ± 0.1, IL-1β + TGFβ1 *vs.* TGFβ1 alone, *P <* 0.05).

### 3.3 Evidence of functional gap junctions between rat adult CFs and CMs following 20 h of co-culture

**Figure 5:**
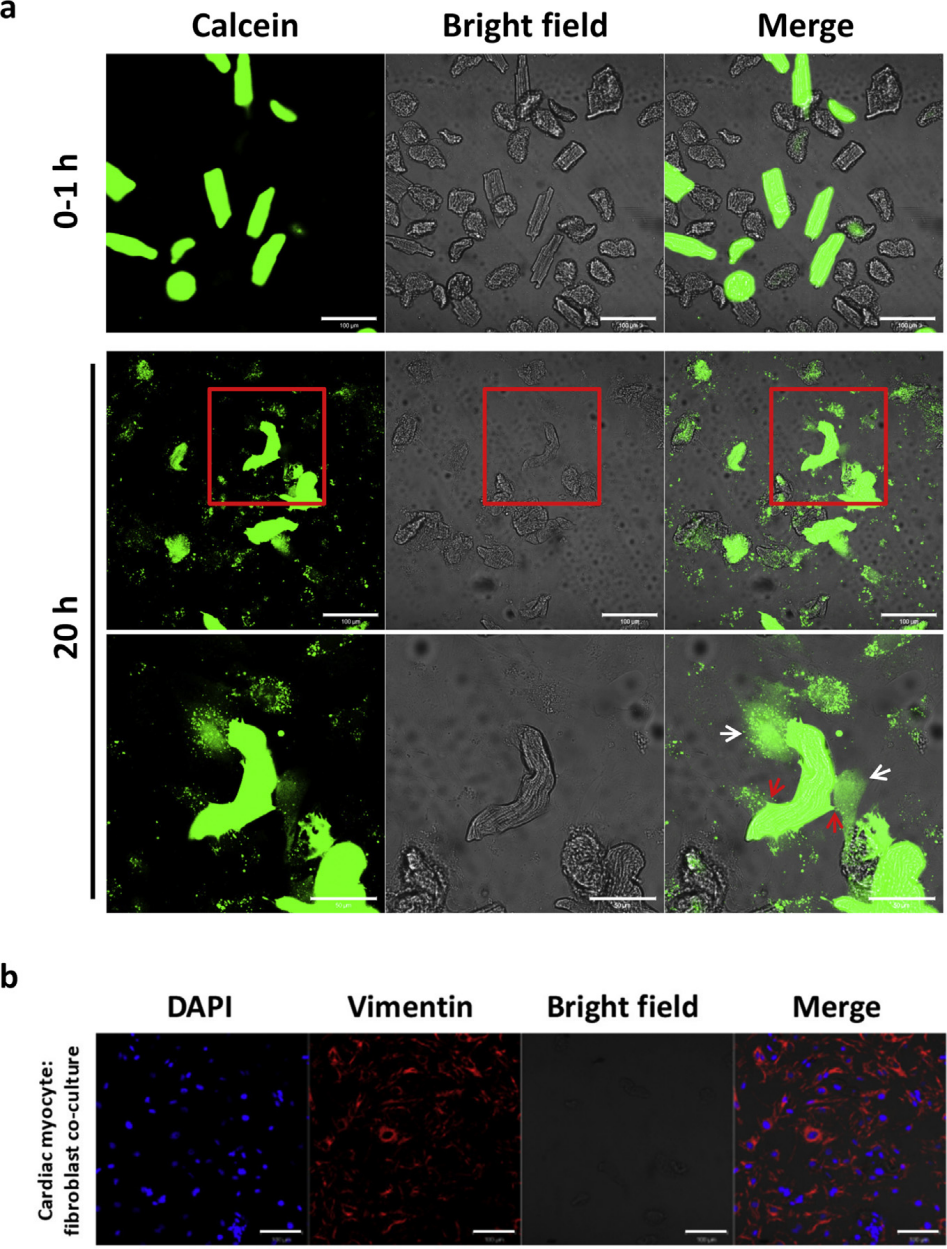
Localisation of calcein in CMs and CFs and vimentin immunofluorescence in co-cultures. (A) Freshly isolated CMs were loaded with 5 μM calcein for 30 min at 37 °C and washed three times in PBS. 1×10^4^ rod shaped CMs in 0.5% FBS media were then added to a CF monolayer in 35 mm glass bottomed petri dishes. The localisation of calcein was visualised immediately following CM addition (0–1 h) then again following 20 h (upper and middle panels respectively). The areas surrounded in the red box in the middle panel are expanded in the bottom panel showing calcein dye transfer to the underlying CFs depicted by white arrows. Red arrows show examples of areas of the CM being pulled by the underlying CFs. Images were taken on the Zeiss 510 LSM confocal microscope. Scale bar represents 100 μm on the upper and middle panels and 50 μm on the lower panel (*n=*3). (B) Cultures were fixed following 24 h following of co-culture and stained for vimentin. All images were taken at x25 magnification on the Zeiss 510 LSM confocal microscope and normalised to an IgG control. Scale bar represents 100 μm (*n=*3).

The presence of functional GJs between adult CMs and CFs was assessed by calcein dye transfer in a co-culture model [8, 9, 10] (Figure 5A). Calcein was confined to CMs immediately following addition of calcein-loaded CMs to CF cultures. However, following 20 h of co-culture, calcein could be detected in the underlying CFs, suggesting the formation of functional heterocellular GJs. Additionally, there were regions of the CM membrane that were being “pulled” by the CFs, suggesting mechanical interactions between the cells (Figure 5A). While is it difficult to see the CF monolayer on the bright field images, vimentin immunofluorescence, following the completion of these experiments, along with the presence of calcein in the CFs, shows the CF monolayer (Figure 5B).

### 3.4 CM contraction duration is shortened in the presence of CFs and this effect is prevented by IL-1β treatment of CFs

**Figure 6:**
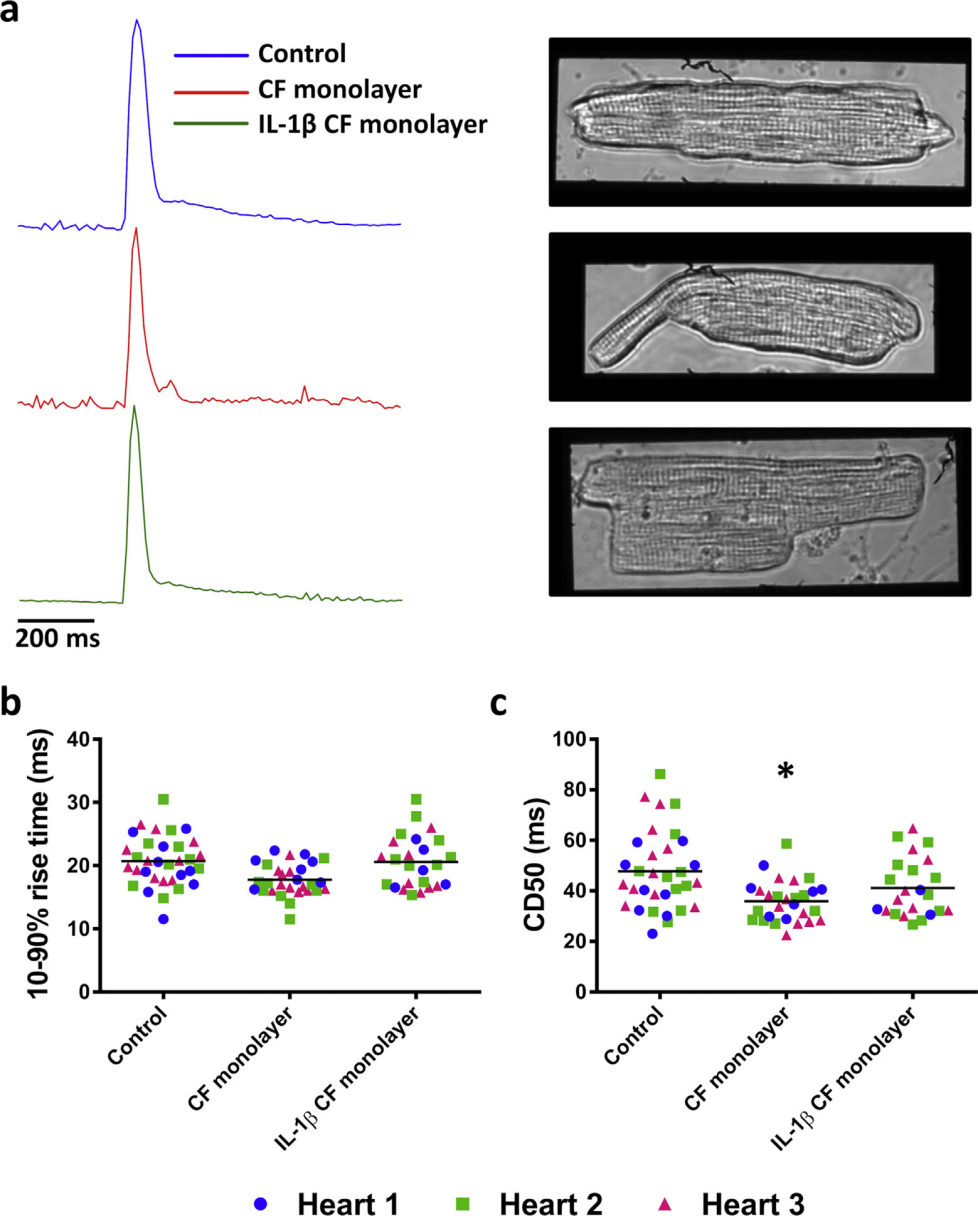
Contraction measurements from CMs cultured alone, with an CF monolayer or with an IL-1β treated CF monolayer. (A) Representative traces and images of CMs cultured alone (top), with a CF monolayer (middle) or with an IL-1β treated CF monolayer (bottom). Scale bar represents 200 ms. (B) 10–90% rise times and (C) CD50 values from CMs from three hearts. Each point represents an individual cell and the mean is denoted by a line. *n=* 33*, n=* 29 and *n=* 25 for control, CF monolayer and IL-1β CF monolayer groups respectively from three animals. *P < 0.05 *vs.* control (one-way repeated measures ANOVA with Tukey's multiple comparison test performed on the mean values from each heart). CD50; time from 50% contraction - 50% relaxation.

CMs in culture alone (control) or in the presence of a CF monolayer +/- IL-1β treatment maintained their rod-like morphology and remained responsive to electrical stimulus (Figure 6A). The CD of CMs cultured alone (control) or in the presence of untreated or IL-1β-treated CFs was examined using a proprietary cell motion algorithm on Image J (Figure 6A). The rise time of the contraction was unaltered between the groups (control, 20.6 ± 0.5 ms; CF monolayer, 17.9 ± 0.9 ms; IL-1β CF monolayer, 20.4 ± 0.7 ms; Figure 6B), while the CD50 was significantly reduced in CMs in co-culture with an untreated CF monolayer (Figure 6C) (36.1 ± 1.1 ms *vs.* 47.3 ± 2.4 ms; CF monolayer *vs*. control; *P* < 0.05). This effect on CM CD50 was lessened when CFs had been pre-treated with IL-1β (39.6 ± 2.6 ms) (Figure 6C)

### 3.5 CFs do not alter the action potential of CMs

**Figure 7:**
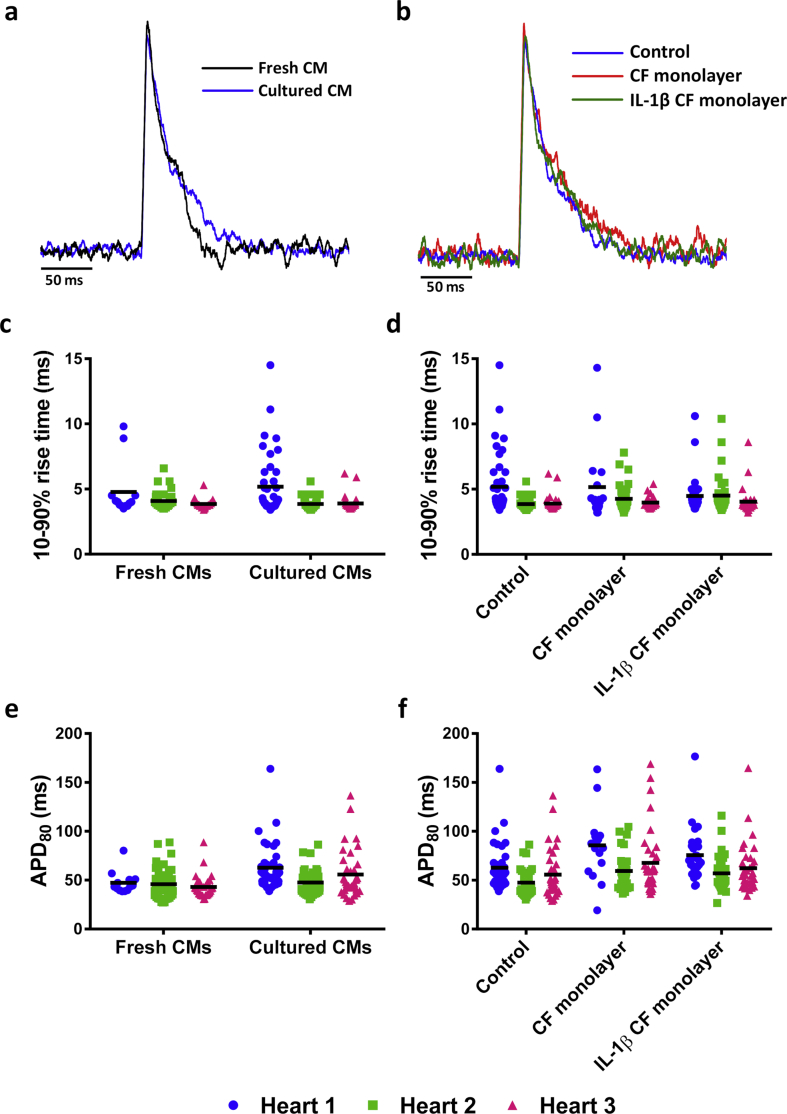
Action potential measurements from fresh CMs or CMs cultured alone, with an CF monolayer or with a IL-1β treated CF monolayer. An overlay of a representative average action potential (AP) from (A) a freshly isolated CM and a CM cultured for 24 h and (B) a CM cultured alone (control) or CMs cultured with a CF monolayer and IL-1β CF monolayer for 24 h (C and D) 10–90% rise time and (E and F) the AP duration of 80% repolarisation (APD80) in respective groups. Scale bar represents 50 ms. Each point represents an individual cell and the mean from each heart is denoted by a line. Values are separated into individual hearts due to the large number of cells. n = 85, n = 122, n = 90 and n = 99 for fresh CMs, cultured CMs/control, CF monolayer and IL-1β CF monolayer groups respectively from three animals. No significant changes were detected between the mean values from each heart using a one-way repeated measures ANOVA with Tukey's multiple comparison test.

Next, APs were measured in freshly isolated CMs or CMs cultured overnight to determine the effect of culture on electrical activity (Figure 7A). There was no change in the 10–90 % rise time (4.2 ± 0.3 ms *vs.* 4.3 ± 0.4 ms, fresh *vs.* cultured CMs; Figure 7C) or APD_80_ (45.5 ± 1.2 *vs.* 55.4 ± 4.4 ms, fresh *vs.* cultured CMs; Figure 7E) between cultured CMs and freshly isolated CMs. Additionally, AP measurements taken from cultured CMs alone (control) or in the presence of an untreated or IL-1β treated CF monolayer (Figure 7B), showed no differences in 10–90 % rise time (control, 4.3 ± 0.4 ms; CF monolayer, 4.5 ± 0.4 ms; IL-1β CF monolayer, 4.3 ± 0.2 ms; Figure 7D) or APD_80_ (control, 55.4 ± 4.4 ms; CF monolayer, 71.0 ± 7.8 ms; IL-1β CF monolayer 65.0 ± 5.5 ms; Figure 7F).

## 4 Discussion

This study set out to examine potential mediators that could be responsible for the enhanced Cx43 expression in CFs from experimental MI hearts. IL-1β was found to be a strong inducer of Cx43 expression in CFs however it was also shown to reduce the MF-like phenotype of the cells. Despite this, they are likely to represent a population of CFs in the damaged heart due to high levels of IL-1β in disease [38]. This expression of Cx43 was shown to be dependent on the IL-1R and downstream NF-κB, p38 MAPK and MEK1 signalling. Furthermore, the effect of co-culture of adult CMs with CFs demonstrated that the CM CD was significantly shortened in comparison to CMs cultured alone. Interesting, when CFs were stimulated with IL-1β this effect on CD was ablated, providing additional evidence that the phenotype of CFs can differentially regulate the function of CMs.

IL-1β induced upregulation of Cx43 levels is in agreement with previous findings in a rabbit synovial fibroblast cell line (HIG-82 cells) [39]. Additionally, stimulation of bladder smooth muscle cells (SMCs) with both IL-1β and TNFα increased Cx43 expression through activation of NF-κB and PKA [40]. NF-κB binding sites have been detected in the Cx43 promoter and have been shown to be important in Cx43 regulation [41]. In the present study, the IL-1β-mediated Cx43 increase in CFs was also found to be dependent on NF-κB, following activation through the IL1R. Furthermore, inhibition of MEK1 significantly blunted Cx43 mRNA upregulation by IL-1β and reduced IL-1β mediated increase in Cx43 protein. Similarly, ERK1/2, which is downstream of MEK1, activation has been shown to promote Cx43 upregulation in various cell types [42, 43]. Conversely, there was no significant effect on Cx43 mRNA expression following inhibition of p38 MAPK or JNK, other known IL-1β target signalling proteins [39]. Protein levels of Cx43 were increased by IL-1β, in the absence but not presence of p38 MAPK and MEK1 inhibitors.

In the current study, CFs in culture were shown to express αSMA: a marker of the MF phenotype. The development of αSMA expression is well established to occur *in vitro* following passage [44, 45]. However, although cultured CFs express αSMA and have some similarities, such as inducing similar reductions on the CM Ca^2+^ transient [45], they have been reported to be dissimilar in phenotype from MFs resident *in vivo* [45, 46]. In the present study, IL-1β was found to significantly reduce the mRNA expression of the MF markers, CTGF and αSMA, as well as significantly inhibiting TGFβ1-mediated increase in CTGF. Similar findings have been reported elsewhere [27, 47].

As well as altering the phenotype of CFs, IL-1β and TGFβ1 are known to have a diverse range of actions following MI. IL-1β is a key driver of the inflammatory phase of cardiac repair, attracting inflammatory cells to the infarct and promoting the release of other proinflammatory mediators [33, 48]. The inflammatory role of TGFβ appears to be more complex with reports of pro- and anti-inflammatory actions, however it is well established to drive healing of the infarct, partly via activation of MFs [49]. Although both IL-1β and TGFβ mRNA have been reported to be enhanced as early as 15 min following MI [50], results from the current study and that of others [27] may suggest that IL-1β acts to suppress TGFβ1-mediated activation of CFs, allowing the infarct to cleared of debris prior to scar formation.

The adult co-culture model utilised in this study allows many interactions between the cells to occur, including those of a paracrine, mechanical and electrical nature, due to media sharing and direct contact allowing the formation of mechanical and GJs. Therefore, it is challenging to interpret what is responsible for the results observed, hence further studies are required to dissect potential mechanisms. Evidence for the formation of both mechanical and GJs was provided in the current study where areas of the CMs were visualised to be “pulled” by underlying CFs and calcein was transferred from CMs to CFs following 20 h of co-culture, respectively.

As control untreated CFs maintained an MF-like phenotype in contrast to IL-1β treated CFs, based on CTGF and SMA detection, this may provide an explanation to the greater effect on CM function following co-culture of CMs with control CFs (MF-like) in comparison to CMs co-cultured with IL-1β-treated CFs (less MF-like). Additionally, mechanical interactions between adult CMs and CFs were observed in our model, which has also been reported by others [10, 51]. As αSMA is part of the cytoskeleton that determines the mechanical properties of cells [27, 52], the levels of αSMA in CFs is likely to influence the mechanical effects on coupled CMs when in co-culture. This may be a contributing factor for the reduction in CD of CMs in co-culture with control CFs, that have greater expression of αSMA, due to an enhanced contractile force and restriction of normal CM contraction. The reduced αSMA in CFs following IL-1β treatment could contribute to less contractile force, consequently elevating contraction restriction of mechanically coupled CMs and effects on CD. Indeed, IL-1β was shown to inhibit TGFβ-mediated contraction of collagen pads by CFs [27], supporting this hypothesis. Furthermore, as CM contraction is reliant on Ca^2+^, changes in contraction likely reflect alterations in Ca^2+^ handling. It is known that the degree of muscle/sarcomere shortening can influence the time course of the Ca^2+^ transient in cardiac muscle [53], resulting from the effect of sarcomere length on Ca^2+^ binding to myofilaments [54]. Thus, the mechanical status of the CMs in co-culture could influence the time course of intracellular Ca^2+^ and therefore CD. However, it must be noted that only mRNA levels of αSMA were examined in the current study and others have reported no change in the protein level following 48 h stimulation with IL-1β, despite reduced levels of mRNA [18]. Therefore, αSMA protein requires evaluation to support this hypothesis. Despite this, a role of αSMA in CF modulating CM function has been previously established, where treating CFs with actin targeting drugs reduced the slowing of CV, ectopic activity and the depolarising effect of CFs in a neonatal CM:CF co-culture model [20].

Although, IL-1β treated CFs had increased expression of Cx43, the CD of CMs was less affected in this condition in comparison to culture with control CFs. This would therefore suggest that the difference in CD was not attributed to Cx43 expression in CFs. However, the degree of coupling and functionality of potential GJs formed was not examined in cultures that had IL-1β-treated CFs.

As well as direct cell-to-cell interactions, the model used in the current study allows for paracrine heterocellular interactions to occur which may also have effects on CM contraction. Indeed, spontaneous contractions of neonatal CMs were inhibited following treatment with fibroblast conditioned media for 72 h, in comparison to control CM cultures which displayed continued contractions [55]. Furthermore, although Ca^2+^ was not measured in the current study, previous work found the Ca^2+^ transient decay to be faster in adult CMs in a CM: CF co-culture transwell model in comparison to CMs alone [45]. This could explain the results of the present study due to quicker uptake/efflux of Ca^2+^ in the presence of CFs, therefore leading to reduced CD. Interestingly, in this previous study, this effect on the Ca^2+^ transient was ablated by TGFβ inhibition, demonstrating a role for TGFβ signalling between the cell types [45].

In the current study, culturing CMs alone or with a CF monolayer (control or IL-1β-treated) had no effect on CM AP rise time or APD_80_. Similarly, culturing swine adult CMs for 24 h showed no significant change in APD_90_, however there was a progressive prolongation over 72 h [56]. Typically, paracrine factors released from CFs have been reported to induce a prolongation in the APD of CMs [6, 15, 57]. Additionally, Cx43 expression in CFs was shown to contribute to the prolonged APD duration observed in neonatal CM: CF co-cultures [13]. In the present study, even after enhanced Cx43 expression, in IL-1β treated CFs, there was no significant alteration in the electrical characteristics of co-cultured CMs in comparison to control CMs. This suggests that any electrical load on CMs as a result of coupling to CFs was not sufficient to change electrical parameters. This is in agreement to a similar co-culture model to the one utilised in the current study with adult rabbit CMs, where there was no difference in APD_90,_ despite sharing of media and evidence for coupling between the cells [9]. The difference to studies involving neonatal CMs is that they have different overall electrical properties [58, 59], therefore may be more easily influenced by coupling to CFs. However, a limitation to this co-culture model is that it is unlikely that all CMs studied were coupled to CFs, therefore differences in well coupled CMs could be diluted and no significant changes detected. Also, a further limitation is that we did not include any positive controls that effect electrical activity in this model.

In summary, our results have shown that IL-1β enhances the expression of Cx43 in CFs through the classical IL-1 signalling pathway. Furthermore, control CFs were demonstrated to alter the function of adult rat CMs by reducing the CD in CMs; an effect that was not apparent when CFs were pre-treated with IL-1β. This demonstrates that the phenotype of CFs is important in heterocellular interactions and further studies are required to dissect the mechanisms responsible for this effect.

## Declarations

### Author contribution statement

L. McArthur: Conceived and designed the experiments; Performed the experiments; Analyzed and interpreted the data; Contributed reagents, materials, analysis tools or data; Wrote the paper.

A. Riddell: Performed the experiments; Analyzed and interpreted the data.

L. Chilton: Conceived and designed the experiments.

S. Nicklin and G. Smith: Conceived and designed the experiments; Contributed reagents, materials, analysis tools or data.

### Funding statement

This work was supported by a British Heart Foundation PhD studentship (FS/12/66/30003).

### Competing interest statement

The authors declare no conflict of interest.

### Additional information

No additional information is available for this paper.

## Appendix A Supplementary data

Supplementary Figure 2 Supplementary Figure 2

Supplementary information_McArthur et al Supplementary information_McArthur et al
