# Supplementary figures and images for: Regulation of connexin 43 by interleukin 1β in adult rat cardiac fibroblasts and effects in an adult rat cardiac myocyte: fibroblast co-culture model
Source: Heliyon. 2019 Dec 30;6(1):e03031. doi: 10.1016/j.heliyon.2019.e03031 (PMC6940628; doi:10.1016/j.heliyon.2019.e03031)

Supplementary figure 1.

**a**

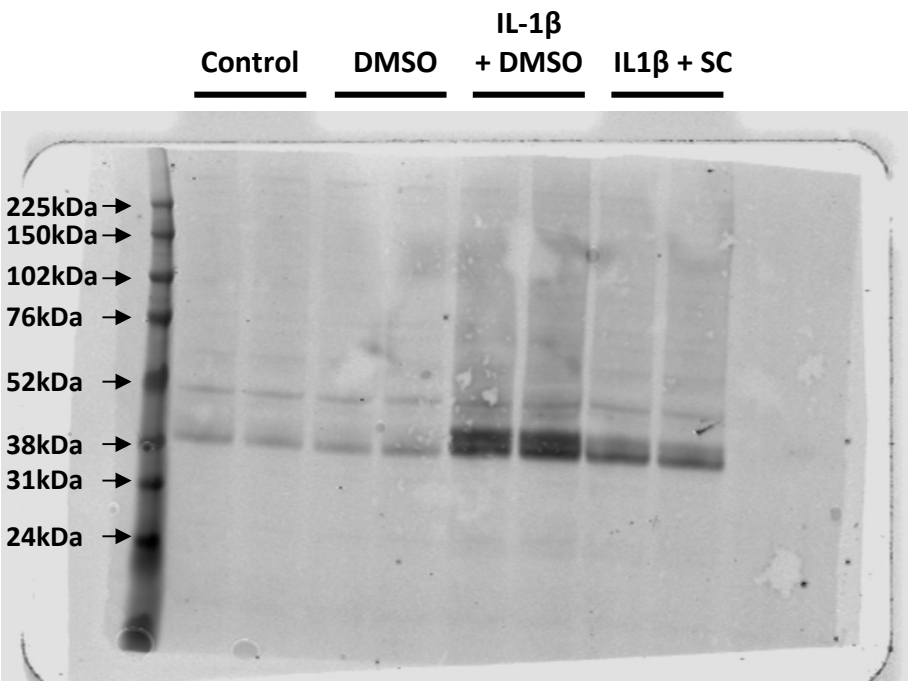

**b**

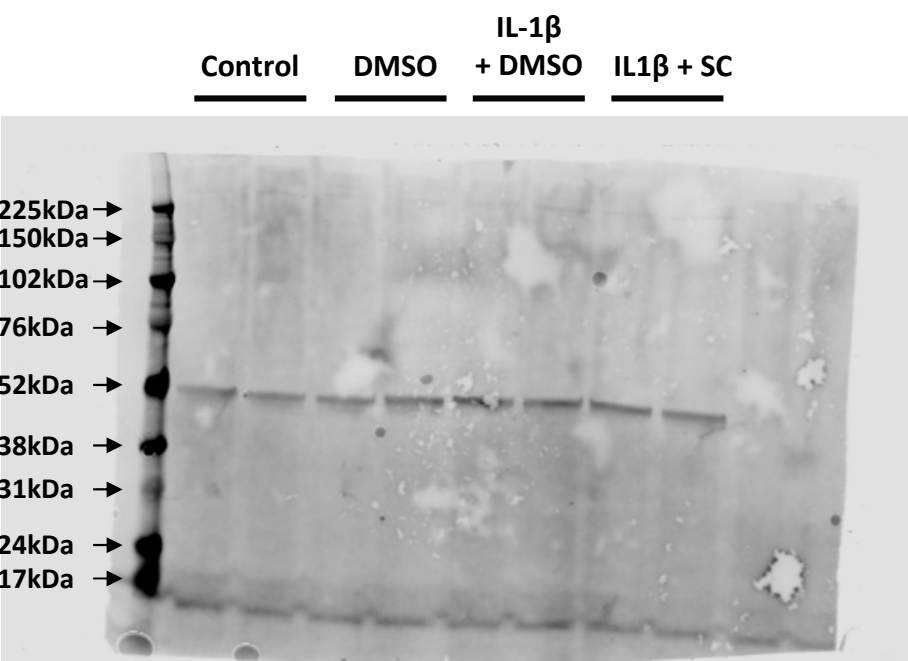

Supplement: Supplementary Figure 1 [file mmc1.pdf]

Supplementary figure 2.

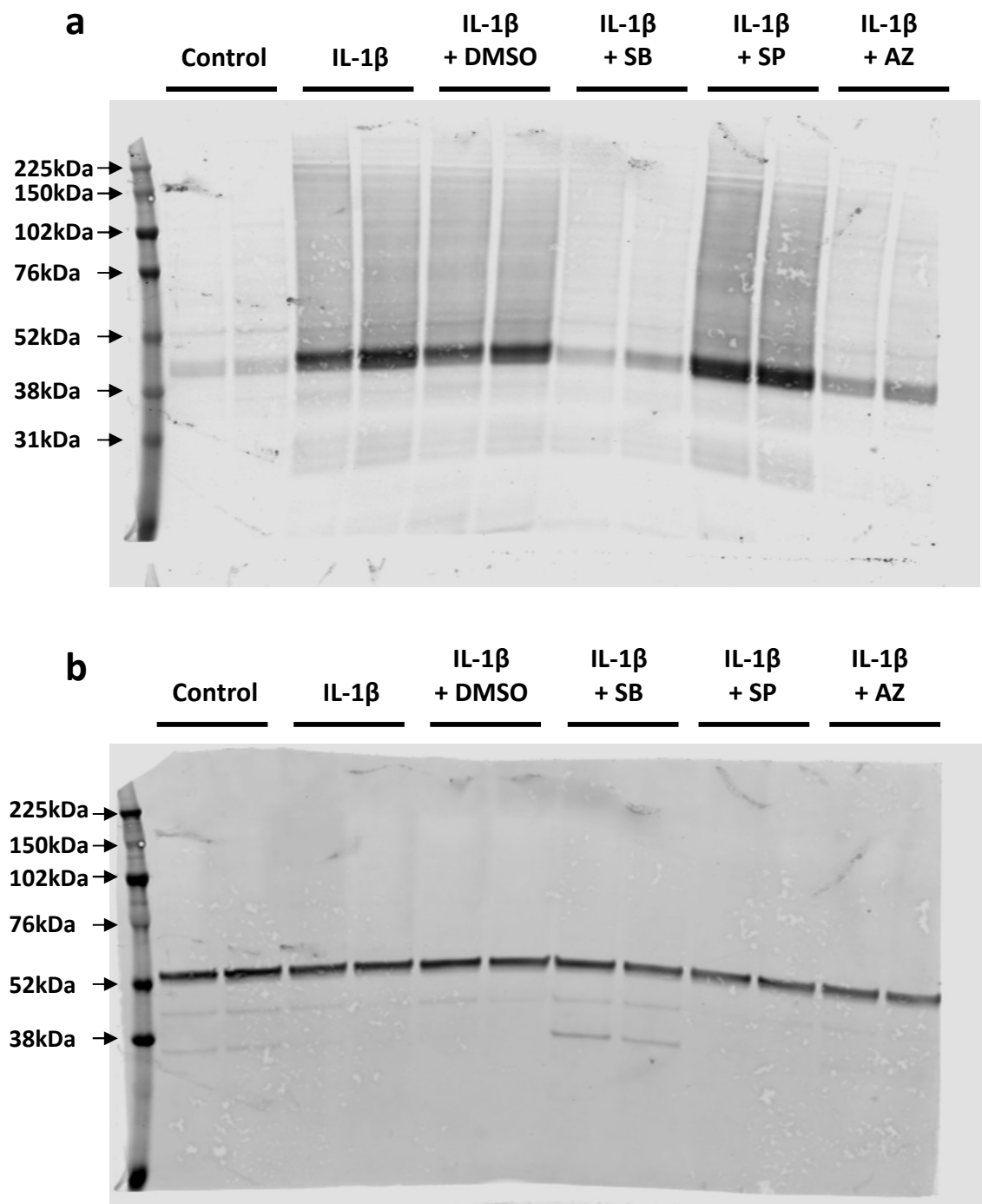

Supplement: Supplementary Figure 2 [file mmc2.pdf]
