# Supplementary material for: Regulation of connexin 43 by interleukin 1β in adult rat cardiac fibroblasts and effects in an adult rat cardiac myocyte: fibroblast co-culture model
Source: Heliyon. 2019 Dec 30;6(1):e03031. doi: 10.1016/j.heliyon.2019.e03031 (PMC6940628; doi:10.1016/j.heliyon.2019.e03031)
Supplement: Supplementary information_McArthur et al [file mmc3.docx]

**Regulation of connexin 43 by interleukin 1β in adult rat cardiac fibroblasts and effects in an adult rat cardiac myocyte: fibroblast co-culture model**

Lisa McArthur, Alexandra Riddell, Lisa Chilton, Godfrey L. Smith, Stuart A. Nicklin*

**Supplementary Methods**

**Isolation and culture of rat adult ventricular cardiac fibroblasts and myocytes**

To isolate CFs, the ventricles were minced in cold ADS buffer prior to transfer to enzyme solution (0.8 mg/mL collagenase type II [Worthington Chemicals, US] and 0.6 mg/mL pancreatin [Sigma] made up in ADS buffer) and incubated at 37°C in a shaking water bath for 10 min. The supernatant from the first digestion was discarded and replaced with fresh enzyme solution prior to another 10 min incubation, shaking at 37°C. A further seven 10 min digestions were then performed followed by a final 30 min digestion in a similar manner. The supernatant containing cells following each digestion was added to FBS, subjected to centrifugation and incubated at 37°C and 5 % CO_2_ following resuspension in FBS.

To isolate CMs, hearts were retrograde perfused with KH heated to 37°C at a constant flow rate of 8 mL/min. Following clearance of blood, the heart was perfused with 0.25 U/mL collagenase type I constituted in a 0.1 % (weight/volume [w/v]) bovine serum albumin (BSA) KH solution. The enzyme was perfused until suitable digestion was completed, which was approximately 12 min. The ventricular tissue was then transferred into the laminar flow cabinet for the remainder of the digestion and minced in Kraft Bruhn buffer (KB) (70.0 mM KOH, 40.0 mM KCl, 50.0 mM L-Glutamic Acid, 20.0 mM Taurine, 20.0 mM KH_2_PO_4_, 3.0 mM MgCl_2_.6H_2_0, 10.0 mM Glucose (Anhydrous), 10.0 mM 4-(2-hydroxyethyl)-1-piperazineethanesulfonic acid (HEPES), 0.5 mM ethylene glycol-bis[β-aminoethyl ether]-N,N,N',N'-tetraacetic acid (EGTA)) containing 1 % (w/v) BSA. Single cells were separated from the undigested tissue and the cell suspension subjected centrifugation for 2 min at 300 × g. CMs were allowed to recover from the digestion in KB buffer for 30 min. Cells were then slowly introduced to increasing concentrations of calcium by incubation in 100 µM followed by 1 mM CaCl in sterile KH for 20 mins per solution. Finally, cells were added to reduced serum media (0.5 % (v/v) FBS).

**qRT-PCR**

RNA was extracted from cells using the QIAzol reagent and the miReasy mini kit (Qiagen) as per manufacturer’s instructions. Total RNA concentration was determined by NanoDrop™ 1000 Spectrophotometer (ThermoFisher Scientific) and RNA reverse transcribed into complementary DNA using Taqman^®^ Reverse Transcription Reagents (ThermoFisher Scientific). qRT-PCR was performed using Taqman assays (ThermoFisher Scientific) for GJA1 (assay ID: Rn01433957_m1), ACTA2 (assay ID: Rn01759928_g1), CTGF (assay ID: Rn01537279_g1) and UBC (assay ID: Rn01499642_m1) as per manufacturer’s instructions. Expression levels of GJA1, ACTA2 and CTGF were normalised to UBC and analysed using the 2^-ΔΔCT^ method.

**Immunofluorescence**

Cells were grown in glass 4-well chamber slides for 48 h prior to fixing with 4% (v/v) paraformaldehyde. To permeabilised cells, 0.1 % Triton X-100 in PBS and incubated for 10 min. Following washes with 0.1 % Tween 20 (PBS-T), cells were incubated in blocking buffer (PBS-T containing 10 [vimentin and αSMA] or 20 % [Cx43] goat serum) for 1 h. The blocking buffer was then removed and primary antibodies diluted as appropriate in blocking buffer were added to the cells. Primary antibodies used (all from Sigma) included mouse monoclonal anti-αSMA (clone IA4, 1:200), mouse monoclonal anti-vimentin (clone LN-6, 1:400) and rabbit polyclonal anti-Cx43 (1:1,000). To control for non-specific antibody binding, some cells were incubated with rabbit IgG or mouse IgG (both from Vector Laboratories, Peterborough, UK) at the same final concentration as the primary antibodies. Primary antibodies and IgGs were incubated overnight at 4°C. The following day cells were washed prior to incubated with an 1:500 dilution (in blocking buffer) of a Alexa Fluro^®^ 488 Goat anti-Rabbit IgG or a Alexa Fluro^®^ 555 Goat anti-Mouse IgG secondary antibody (ThermoFisher Scientific) for primary antibodies raised in rabbit and mouse, respectively. Secondary antibodies were incubated for 1 hr protected from light. Cells were then washed prior to mounting with ProLong^®^ Gold Antifade Mountant with 4',6-diamidino-2-phenylindole (DAPI) (ThermoFisher Scientific) to counterstain nuclei. Slides were imaging on an LSM 510 Meta laser scanning confocal microscope using LSM510 software (Zeiss, UK).

Quantification of Cx43 immunofluorescence was conducted on at least two images per condition from each experiment using the Image J threshold tool. All pixels where staining was present were highlighted and fluorescence arbitrary units normalised to the total cell number, as determined by the DAPI counterstain, in the respective images. Control images were analysed first and the same settings used for IL-1β treated cells for each experiment.

**Immunoblotting**

Cells were lysed using a lysis buffer (50.0 mM Tris-HCl, 50.0 mM NaF, 1.0 mM Na_4_P_2_O_7_-10H_2_O, 1.0 mM ethylenediaminetetraacetic acid [EDTA] and 1 mM EGTA) containing 1 % (v/v) Triton X-100, 250 mM mannitol, 1 mM dithiothreitol (DTT), 1 mM Na_3_VO_4_, 0.1 mM phenylmethylsulfonyl fluoride (PMSF) and complete protease inhibitor cocktail (Roche Diagnostics). Protein concentrations were determined using a Pierce™ BCA Protein Assay Kit (ThermoFisher Scientific), as per manufacturer’s instructions. Cell lysates were fractioned by SDS-PAGE and transferred to a 0.2 µm nitrocellulose membrane (GE Healthcare Life Sciences, UK). Membranes were blocked in blocking buffer (TBS‑T: SEA BLOCK blocking buffer [ThermoFisher Scientific]) at a ratio of 1:1) for 1 h at room temperature. Membranes were then incubated with blocking buffer containing the primary antibodies, rabbit polyclonal anti-Cx43[1] (1: 5,000) and mouse monoclonal anti-β-tubulin[1] (clone AA2, Sigma, 1: 2500) overnight at 4°C on a shaker. To detect antibody binding, membranes were incubated for 1 h at room temperature on a shaker with a 1:15,000 dilution of IRDye^®^ 680RD Goat anti-Mouse IgG (H + L) secondary antibody (LI‑COR Biosciences, Cambridge, UK) and Alexa Fluro^®^ 790 Goat anti-Rabbit IgG secondary antibody (ThermoFisher Scientific) in blocking buffer. Protein bands were imaged on the Odyssey^®^ CLx Imaging System (LI‑COR Biosciences) using the appropriate channel and quantified using Image Studio Lite v5 Software.

**Contraction measurement**

The Image J macro used to examine contraction duration (CD) measures optical differences from a reference frame (typically frame 1). This indirect measure of contraction was plotted as the sum of absolute difference (SAD) against time.

**Action potential measurements**

To prepare cells for AP measurement, a 1:6,000 dilution of FluoVolt™ in media was added to the cells and incubated for 25 min at 5 % CO_2_ and 37°C prior to the replacement of fresh media. Loading of the cells with FluoVolt^TM^ in each plate was staggered so that the recordings were taken within 20 min of loading. Cardiac muscle cells were chosen at random and a shutter positioned around an individual cardiac cell using HCImage Live software to restrict the area of recording and reduce noise in the trace.

References

[1] S.R. Johnstone, A.K. Best, C.S. Wright, B.E. Isakson, R.J. Errington, P.E. Martin, Enhanced connexin 43 expression delays intra-mitotic duration and cell cycle traverse independently of gap junction channel function, Journal of cellular biochemistry 110(3) (2010) 772-82.

**Supplementary figures captions**

**Supplementary figure ‎1. Full length blots of cropped image in Figure 2C.** (A) Cx43 and (B) β-tubulin detection.

**Supplementary figure ‎2. Full length blots of cropped image in Figure 2E.** (A) Cx43 and (B) β-tubulin detection.
